# Vitamin D, Folic Acid and Vitamin B_12_ Can Reverse Vitamin D Deficiency-Induced Learning and Memory Impairment by Altering 27-Hydroxycholesterol and S-Adenosylmethionine

**DOI:** 10.3390/nu15010132

**Published:** 2022-12-27

**Authors:** Lijing Wang, Cui Zhou, Huiyan Yu, Ling Hao, Mengwei Ju, Wenjing Feng, Zhiting Guo, Xuejing Sun, Qiushi Fan, Rong Xiao

**Affiliations:** 1 Beijing Key Laboratory of Environmental Toxicology, School of Public Health, Capital Medical University, Beijing 100069, China; 2 Medical Nutrition, College of Allied Health Professions, University of Nebraska Medical Center, Omaha, NE 68198, USA

**Keywords:** vitamin D, 27-hydroxycholesterol, CYP27A1, S-Adenosylmethionine, memory, Alzheimer’s disease

## Abstract

The cholesterol-oxidized metabolite 27-hydroxycholesterol (27-OHC) is synthesized by CYP27A1, which is a key factor in vitamin D and oxysterol metabolism. Both vitamin D and 27-OHC are considered to play important roles in Alzheimer’s disease (AD). The study aims to research the effects of co-supplementation of vitamin D, folic acid, and vitamin B_12_ on learning and memory ability in vitamin D-deficient mice, and to explore the underlying mechanism. In this study, C57BL/6J mice were fed a vitamin D-deficient diet for 13 weeks to establish a vitamin D-deficient mice model. The vitamin D-deficient mice were then orally gavaged with vitamin D (VD), folic acid (FA), and vitamin B_12_ (VB_12_) alone or together for eight weeks. Following the gavage, the learning and memory ability of the mice were evaluated by Morris Water Maze and Novel object recognition test. The CYP27A1-related gene and protein expressions in the liver and brain were determined by qRT-PCR. The serum level of 27-OHC was detected by HPLC-MS. Serum levels of 25(OH)D, homocysteine (Hcy), and S-Adenosylmethionine (SAM) were measured by ELISA. After feeding with the vitamin D-deficient diet, the mice performed longer latency to a platform (*p* < 0.001), lower average speed (*p* = 0.026) in the Morris Water Maze, a lower time discrimination index (*p* = 0.009) in Novel object recognition, and performances were reversed after vitamin D, folic acid and vitamin B_12_ supplementation alone or together (*p* < 0.05). The gene expressions of CYP27A1 in the liver and brain were upregulated in the vitamin D-deficiency (VDD) group compared with the control (CON) group (*p* = 0.015), while it was downregulated in VDD + VD and VDD + VD-FA/VB_12_ groups compared with the VDD group (*p* < 0.05), with a similar trend in the protein expression of CYP27A1. The serum levels of 27-OHC were higher in the VDD group, compared with CON, VDD + VD, and VDD + VD-FA/VB_12_ group (*p* < 0.05), and a similar trend was found in the brain. The serum 25(OH)D levels were significantly decreased in the vitamin D-deficiency group (*p* = 0.008), and increased in the vitamin D-supplemented group (*p* < 0.001). The serum levels of SAM were higher in the B vitamins-supplemented group, compared with CON and VDD groups (*p* < 0.05). This study suggests that CYP27A1 expression may be involved in the mechanism of learning and memory impairment induced by vitamin D deficiency. Co-supplementation with vitamin D, folic acid, and vitamin B_12_ significantly reverses this effect by affecting the expression of CYP27A1, which in turn regulates the metabolism of 27-OHC, 25(OH)D, and SAM.

## 1. Introduction

An increasing number of studies have proved that vitamin D deficiency may play an important role in the pathological progression of Alzheimer’s disease (AD) [1,2]. Vitamin D is a micronutrient that is essential to brain development and function, such as synaptic plasticity, neurotransmission, and neuroprotection [3]. A dose-response meta-analysis revealed that vitamin D deficiency was associated with a higher risk of AD, and adequate levels of vitamin D concentration were related to a lower risk of AD [4]. However, due to the limitation of only a few studies on this topic, the risk of AD induced by vitamin D deficiency may be overestimated. Similarly, an animal study reported that vitamin D deficiency accelerated cognitive impairment via increasing amyloid plaque (Aβ) deposition, inflammatory factor secretion, and tau phosphorylation [5]. Therefore, vitamin D deficiency is recognized as being related to AD. However, the pathology of how vitamin D deficiency induces AD remains unclear and needs to be elucidated.

As one of the important components of the brain, cholesterol plays an important role in maintaining normal brain functions. Increasingly, studies have confirmed that impaired cholesterol homeostasis is involved in the development of AD, epidemiological evidence revealed that individuals with a high level of blood cholesterol are more likely to develop AD, and animal experiments showed that high cholesterol diets induced AD-like pathology [6]. However, due to the existence of the blood-brain barrier, it still cannot reveal the mechanism of AD caused by cholesterol. 27-OHC, synthesized from cholesterol, is the main peripheral oxysterol that could cross the blood-brain barrier and take part in the pathogenesis of AD [7]. Much epidemiology evidence has demonstrated that high levels of 27-hydroxycholesterol (27-OHC) were associated with memory deficits and AD [8,9]. A previous study has found that high levels of dietary cholesterol promoted the production of 27-OHC in the brain and further impaired learning and memory ability, yet, inhibition of 27-OHC production could reverse the impairment [10]. Therefore, 27-OHC may be a key factor in AD pathogenesis.

CYP27A1 is an enzyme involved in the activation of vitamin D and the production of 27-OHC. Vitamin D can be activated by the enzyme CYP27A1 to produce 25-hydroxyvitamin D (25(OH)D), which is then activated by the enzyme CYP27B1 to produce 1alpha,25-dihydroxyvitamin D (1,25(OH)2D) [11,12]. Meanwhile, 27-OHC is a key metabolite of cholesterol after degradation mainly by the enzyme CYP27A1, which has been proven to be associated with AD [13]. An animal study indicated that 27-OHC elevation negatively impacts bone homeostasis, also, vitamin D deficiency can result in bone homeostasis disorder [14,15]. Since CYP27A1 participates in both 27-OHC synthesis and vitamin D signaling pathways, whether vitamin D deficiency affects learning and memory ability by regulating CYP27A1 expression and 27-OHC metabolism, remains unknown. 

In addition to vitamin D, folic acid and vitamin B_12_ also play important roles in AD. Recently, a case-control study investigated serum vitamins and the risk of AD in the Chinese population and found that lower levels of vitamin D and B vitamins were associated with higher risks of AD, especially folic acid and vitamin B_12_ [16]. A meta-analysis also demonstrated that B vitamins supplementation is related to the slowing of cognitive function decline, suggesting that the population at risk of cognitive impairment should maintain an adequate B vitamin status [17]. Homocysteine (Hcy) levels, relating to the risk of AD, will be increased when folic acid and vitamin B_12_ are inadequate [18]. Folic acid and vitamin B_12_ participate in the formation of S-Adenosylmethionine (SAM), which is the methyl donor for methylation reactions and may improve cognitive function in AD [19]. A retrospective study found that serum homocysteine (Hcy) levels decreased with increasing vitamin D levels, which provided a perspective that vitamin D, folic acid, and vitamin B_12_ co-supplementation may be better than either alone [20]. The synergistic effects and mechanism of vitamin D, folic acid, and vitamin B_12_ co-supplementation in improving cognitive function need to be further explored, as the three vitamins alone show a limited improvement in cognitive function and remain controversial.

Hence, we hypothesized that vitamin D deficiency may lead to a decline in learning and memory ability by affecting CYP27A1. Vitamin D intervention can downregulate the expression of CYP27A1, then reduce the levels of 27-OHC, which then reverses the decline in learning and memory ability induced by vitamin D deficiency. Besides, folic acid and vitamin B_12_ co-supplementation may play a joint role in the process by regulating the levels of Hcy and SAM. The results of this study may provide a new direction for the discovery of the mechanism involved in vitamin D deficiency and AD. 

## 2. Materials and Methods

### 2.1. Animals and Models

Male C57BL/6J mice used in this study were around 8 months old and were purchased from Animal Research Center of Capital Medical University (Beijing, China). The mice were housed in a SPF facility with light/dark cycles (12 h/12 h), temperature-controlled (22 ± 2 °C), with free access to water. All animal experiments were performed after receiving approval from the ethics committee of Capital Medical University (Ethics approval code: AEEI-2019-175). 

**Figure 1:**
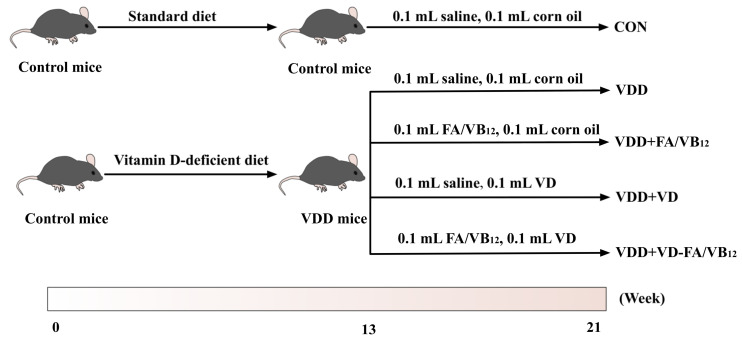
The flow chart. CON: control; VDD: vitamin D-deficient diet; VDD + FA/VB_12_: vitamin D-deficient diet plus folic acid/vitamin B_12_ supplement; VDD + VD: vitamin D-deficient diet plus vitamin D supplement; VDD + VD-FA/VB_12_: vitamin D-deficient diet plus vitamin D and folic acid/vitamin B_12_.

After adaptive feeding for 2 weeks, forty mice were randomly and equally divided into five groups based on initial body weights evenly. The five groups are as follows: Control group (CON), Vitamin D deficient group (VDD), Vitamin D deficient and folic acid/vitamin B_12_ supplemented group (VDD + FA/VB_12_), Vitamin D deficient and vitamin D supplemented group (VDD + VD), Vitamin D deficient and vitamin D, folic acid/vitamin B_12_ supplemented group (VDD + VD-FA/VB_12_). Control mice were fed an AIN93 standard diet, and VDD mice were fed a vitamin D-deficient diet for 13 weeks to establish a vitamin D-deficient mice model [5]. Following this, the mice were supplemented with cholecalciferol (1000 IU/kg), folic acid (13 mg/kg), and vitamin B_12_ (80 ug/kg), respectively, by oral gavage, the control group was maintained with the same volumes of saline and corn oil, four times a week and this lasted for 8 weeks. The flow chart is shown in Figure 1. The vitamin D-deficient diet compositions were shown in Supplementary Table S1. The dose of supplement was according to the study published previously [21]. After 21 weeks of intervention, behavioral tests were conducted, then the mice were anesthetized followed by collecting the blood samples and fresh tissues. All of the samples were then frozen at −80 °C until use. 

### 2.2. Morris Water Maze Test

Morris water maze test was used to determine the spatial learning and memory ability of the mice [22]. A white circular pool was divided into four equal virtual quadrants filled with water, and a platform was fixed in the center of a targeted quadrant beneath the water. The experiment was divided into two procedures: place navigation and spatial probe. First, the mice were allowed to swim from three quadrants for 90 s one time, and they would be guided to remain on the platform for 10 s if they had escaped onto the platform. After five days of training, the platform was taken away, and the mice were released from the furthest quadrant and allowed to swim freely for 90 s, to conduct the spatial probe on the sixth day. The time of escape latency, mean distance to platform, average speed, number of platform-site crossovers, and retention time in target quadrant were recorded.

### 2.3. Novel Object Recognition Test

Novel object recognition was used to detect the short-term memory of mice [23]. In the habituation phase, the mice were put into a 50 cm × 50 cm × 40 cm apparatus for 5 min without objects on the first day, 70% ethanol was used to eliminate the residual odor. Then the test was split into two sessions: on the second day, the mice were placed into apparatus containing two identical objects for 10 min; on the third day, one familiar object was substituted with a novel one, and the mice were placed into apparatus for 10 min. Exploration behavior was defined as directing attention to the object no more than 2 cm or touching the object. Time discrimination index (TDI) was calculated as (the time exploring the novel object- the time exploring the familiar object)/(the time exploring the familiar object + the time exploring the novel object). Frequency discrimination index (FDI) was calculated as (the frequency exploring the novel object- the frequency exploring the familiar object)/(the frequency exploring the familiar object + the frequency exploring the novel object).

### 2.4. High-Performance Liquid Chromatography-Mass Spectrometry (HPLC-MS)

The concentration of 27-OHC in serum and brain was measured using HPLC–MS [24]. In brief, 50 µL serum or brain homogenization was added to 2 mL of chloroform. 27-hydroxycholesterol with a concentration of 50 ng/mL was added to the calibration standards as an internal standard. Acidic buffer and methyl tert-butyl was added to each tube followed by vortex. All of the extracts were stored at −80 °C for 60 min. The extracts were taken to dryness under nitrogen, then derivatization reagent was added and shaken for 90 min. The mixture was then centrifuged and put into nitrogen evaporator, centrifuged at 13,000× *g* for 10 min. Finally, a total of 75 µL sample was injected into the HPLC-MS system and the quantification of 27-OHC was performed.

### 2.5. Enzyme-Linked Immunosorbent Assay (ELISA)

The serum levels of 25(OH)D, Hcy, and SAM were detected by ELISA according to previously published research [25,26,27]. Blood samples were centrifuged at 3000× *g* for 15 min at 4 °C and stored at −80 °C until measurement. Then the ELISA kits were used to measure serum 25(OH)D (ImmunoDiagnostic Systems, Inc, Tyne and Wear, UK), serum Hcy (Cusabio, Wuhan, China), and serum SAM (Cloud clone Corp, Houston, TX, USA) in accordance with the manufacturers’ instructions.

### 2.6. Quantitative Real-Time PCR (qRT-PCR)

The total mRNA was extracted from livers and brains by using the SV Total RNA Isolation System (Promega, Madison, WI, USA) followed by reverse transcription using the Reverse Transcription System (Promega, Madison, WI, USA). qRT-PCR was used to analyze the mRNA expression levels of CYP27A1, CYP27B1and VDR in liver and brain. Primers were designed specifically based on the NCBI database, the forward and reverse primer sequences used in this study are shown in Supplementary Table S2. qRT-PCR reactions were performed following the protocols using a CFX Connect Real-Time PCR Detection System (Bio-Rad, Hercules, CA, USA). The gene β-actin served as an internal reference for normalization.

### 2.7. Western Blot

Liver or brain tissues were lysed with RIPA buffer containing protease inhibitor (PMSF) on ice for 30 min and centrifuged at 12,000× *g* for 10 min at 4 °C. The supernatant was reserved and the protein concentrations were determined with BCA assay. An equal amount of protein was separated through SDS-PAGE running and then transferred to PVDF membranes (Millipore, Boston, MA, USA). The antibodies used were as follows: CYP27A1 1:5000 (Abcam, Boston, MA, USA), CYP27B1 1:1000 (Abcam, Boston, MA, USA), VDR 1:1000 (CST, Boston, MA, USA), β-actin 1:5000 (Abcam, Boston, MA, USA), and Goat anti-rabbit IgG 1:3000 (CST, Boston, MA, USA). Fluorchem FC 2 software was used to analyze the image and the relative expression of target proteins and normalized to β-actin protein.

### 2.8. Statistical Analysis

SPSS 23.0 and GraphPad Prism 9.1.1 were used for statistical analysis. All data were expressed as mean ± standard error (mean ± SEM). One-way ANOVA (analysis of variance) and least significance difference post-hoc test were used to evaluate the significant differences between groups. Two-way ANOVA analysis was used for repeated measurement data in Morris water maze test. *p* < 0.05 was considered to be statistically significant.

## 3. Results

### 3.1. Long-Term Spatial Memory Performance via Morris Water Maze

**Figure 2:**
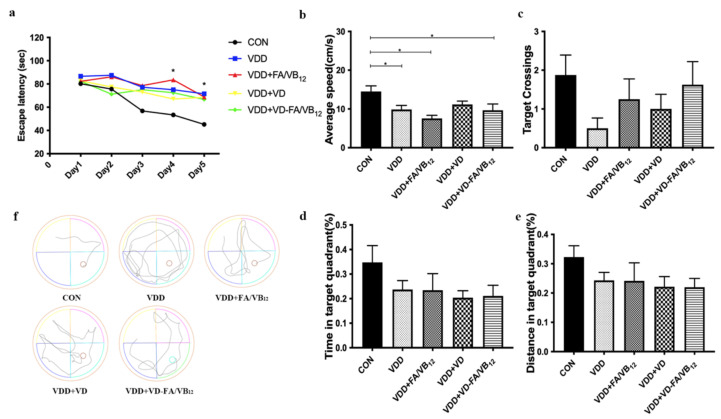
Spatial learning and memory ability in mice via Morris water maze. (**a**) Escape latency; (**b**) Average speed; (**c**) Number of platform-site crossovers; (**d**) Time in the target quadrant; (**e**) Distance in the target quadrant; (**f**) Tracks of probe trial. CON: control; VDD: vitamin D-deficient diet; VDD + FA/VB_12_: vitamin D-deficient diet plus folic acid/vitamin B_12_ supplement; VDD + VD: vitamin D-deficient diet plus vitamin D supplement; VDD + VD-FA/VB_12_: vitamin D-deficient diet plus vitamin D and folic acid/vitamin B_12_. All data are presented as the mean ± SEM (*n* = 8). A two-sided *p* < 0.05 was considered statistically significant.

The Morris water maze test was used to assess the long-term spatial memory of mice. Results are shown in Figure 2. The escape latency decreased significantly in all groups of mice as the training time increased (F = 9.913, *p* < 0.001, Figure 2a). Besides, significant differences in escape latency among groups also appeared on the last two days as shown in Figure 1a (F = 9.206, *p* < 0.001). Compared with the CON group, the escape latency of the VDD (*p* < 0.001), VDD + FA/VB_12_ (*p* < 0.001), VDD + VD (*p* = 0.001), and VDD + VD-FA/VB_12_ (*p* = 0.001) groups were significantly increased on day four and dayfive. On day four, the escape latency of the mice in the VDD group (*p* = 0.035) and VDD + FA/VB_12_ (*p =* 0.002) group, but not the VDD + VD group and VDD + VD-FA/VB_12_ group, were significantly longer than the CON group. On day five, only the VDD group (*p* = 0.036) had a higher escape latency compared to the CON group. There were no interaction effects between factors of training time and group in the training period (F = 1.066, *p* = 0.385). The average speed of mice was different among groups (*p* = 0.019), significantly slower speeds were observed in the VDD (*p* = 0.049), VDD + FA/VB_12_ (*p* = 0.001), and VDD + VD-FA/VB_12_ groups (*p* = 0.014) in comparison to the CON group (Figure 2b). No difference was found in platform-site crossovers, time, and distance in the target quadrant (all *Ps p* > 0.05) (Figure 2c–e). The results indicated that the impaired long-term spatial memory led by vitamin D deficiency may be restored by the intervention of vitamin D and B vitamins.

### 3.2. Short-Term Memory via Novel Object Recognition

**Figure 3:**
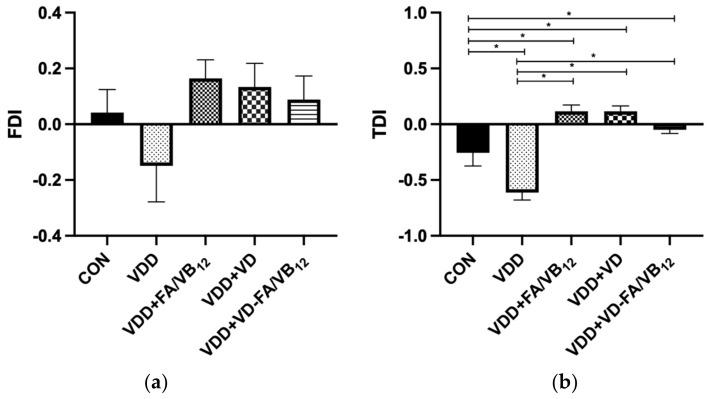
Short-term memory in mice tested by novel object recognition. (**a**) FDI; (**b**) TDI; CON: control; VDD: vitamin D-deficient diet; VDD + FA/VB_12_: vitamin D-deficient diet plus folic acid/vitamin B_12_ supplement; VDD + VD: vitamin D-deficient diet plus vitamin D supplement; VDD + VD-FA/VB_12_: vitamin D-deficient diet plus vitamin D and folic acid/vitamin B_12_; FDI: frequency discrimination index; TDI: time discrimination index. All data are presented as the mean ± SEM (*n* = 8). A two-sided *p* < 0.05 was considered statistically significant.

The novel object recognition test was performed to measure the short-term memory of the mice. Details of the results are shown in Figure 3. There were significant differences in the TDI of mice among groups (F = 18.483, *p* < 0.001). Compared with the CON group, the TDI of the VDD group (*p* = 0.001) was significantly decreased, while VDD + FA/VB_12_ (*p* = 0.001), VDD + VD (*p* = 0.001), and VDD + VD-FA/VB_12_ (*P* = 0.048) groups revealed significant increased TDI. Mice in VDD + FA/VB_12_, VDD + VD, and VDD + VD-FA/VB_12_ groups had higher TDI than that in the VDD group (*p* < 0.001). Additionally, although no significant difference was found in FDI among groups, VDD + FA/VB_12_, VDD + VD and VDD + VD-FA/VB_12_ groups showed higher FDI than the VDD group. These results revealed that the supplement of vitamin D and/or B vitamins reversed the decline in short-term memory due to vitamin D deficiency.

### 3.3. Vitamin D and B Vitamins Related Factors in Serum

**Figure 4:**
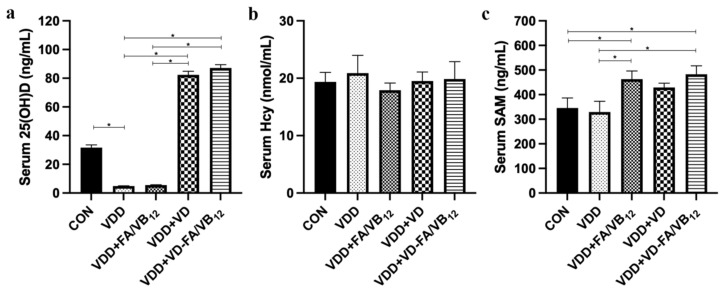
Vitamin D and B vitamins related metabolism levels in serum. (**a**) Serum 25(OH)D level; (**b**) Serum Hcy level; (**c**) Serum SAM level. CON: control; VDD: vitamin D-deficient diet; VDD + FA/VB_12_: vitamin D-deficient diet plus folic acid/vitamin B_12_ supplement; VDD + VD: vitamin D-deficient diet plus vitamin D supplement; VDD + VD-FA/VB_12_: vitamin D-deficient diet plus vitamin D and folic acid/vitamin B_12_. All data are presented as the mean ± SEM (*n* = 6). A two-sided *p* < 0.05 was considered statistically significant.

To investigate the mechanisms by which vitamin D and B vitamins restore memory capacity in mice, we measured the levels of their related factors in the serum. As shown in Figure 4, compared to the CON group, the serum level of 25(OH)D was significantly decreased in the VDD group (*p* = 0.002). The serum 25(OH)D levels of the VDD + VD group and VDD + VD-FA/VB_12_ group were significantly higher than the VDD group and VDD + FA/VB_12_ group (*p* < 0.001). The serum Hcy level of the VDD group was higher than the other groups and VDD + FA/VB_12_ group was lower, however, the differences were not statistically significant. The serum level of SAM showed a significant increase in the VDD + FA/VB_12_ group and VDD + VD-FA/VB_12_ group compared with the CON group (*p* = 0.027, *p* = 0.011) and VDD group (*p* = 0.013, *p* = 0.005). These results suggested that vitamin D supplementation increased the serum 25(OH)D concentrations in all treated mice. B vitamins supplementation alone or in combination with vitamin D also increased serum SAM levels.

### 3.4. 27-OHC Level in Serum and Brain

**Figure 5:**
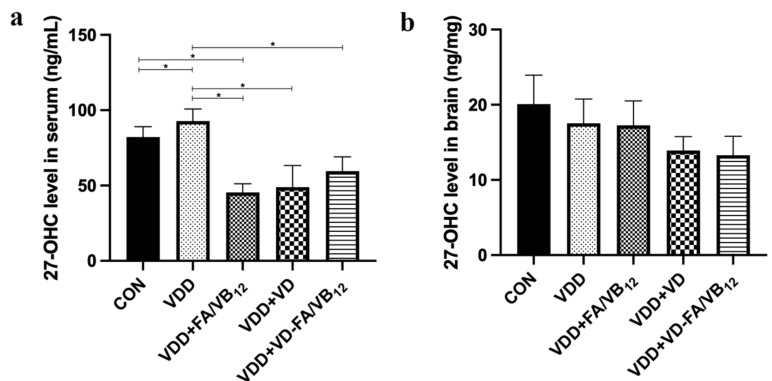
27-OHC concentration in serum and brain. (**a**) 27-OHC level in serum; (**b**) 27-OHC level in brain. CON: control; VDD: vitamin D-deficient diet; VDD + FA/VB_12_: vitamin D-deficient diet plus folic acid/vitamin B_12_ supplement; VDD + VD: vitamin D-deficient diet plus vitamin D supplement; VDD + VD-FA/VB_12_: vitamin D-deficient diet plus vitamin D and folic acid/vitamin B_12_. All data are presented as the mean ± SEM (*n* = 3). A two-sided *p* < 0.05 was considered statistically significant.

In order to investigate the mechanism of memory improvement in vitamin D-deficient mice by vitamin D and/or B vitamins co-supplementation, the concentration of 27-OHC in serum and brain was measured for its important role in the progress of AD. As shown in Figure 5, compared to the CON group, the serum level of 27-OHC was significantly increased in the VDD group (*p* = 0.020) and decreased in VDD + FA/VB_12_ group (*p* = 0.030). The 27-OHC concentration in VDD + FA/VB_12_ group (*p* = 0.005), VDD + VD group (*p* = 0.008), and VDD + VD-FA/VB_12_ group (*p* = 0.031) was significantly decreased when compared with that in the VDD group. The 27-OHC level in the brain performed a similar trend to that in serum, although it was not statistically significant. The above results demonstrated that the supplementation of vitamin D and/or B vitamins may inhibit the increase of 27-OHC induced by vitamin D deficiency.

### 3.5. Expressions of CYP27A1, CYP27B1 and VDR in Liver and Brain

**Figure 6:**
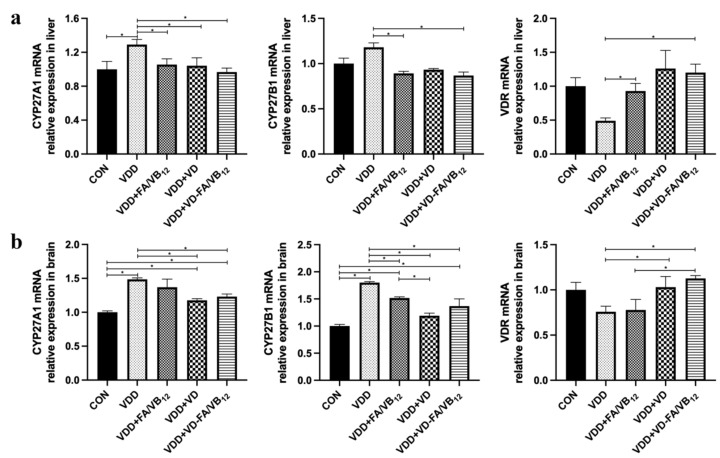
The gene expression of CYP27A1, CYP27B1, and VDR in liver and brain. (**a**) CYP27A1, CYP27A1and VDR mRNA in livers; (**b**) CYP27A1, CYP27A1and VDR mRNA in brains. CON: control; VDD: vitamin D-deficient diet; VDD + FA/VB_12_: vitamin D-deficient diet plus folic acid/vitamin B_12_ supplement; VDD + VD: vitamin D-deficient diet plus vitamin D supplement; VDD + VD-FA/VB_12_: vitamin D-deficient diet plus vitamin D and folic acid/vitamin B_12_. All data are presented as the mean ± SEM (*n* = 4). A two-sided *p* < 0.05 was considered statistically significant.

Vitamin D and 27-OHC metabolism-related gene expressions in the liver and brain were significantly different among groups. Results are shown in Figure 6. Compared with the CON group, the mRNA expression of CYP27A1 (*p* = 0.015) was upregulated, while the VDR (*p* = 0.032) was downregulated in the liver of mice in the VDD group. Compared with the VDD group, the mRNA expression of CYP27A1 and CYP27B1 were downregulated in the VDD + FA/VB_12_ group (*p* = 0.041, *p* = 0.050), VDD + VD group (*p* = 0.033, *p* = 0.558) and VDD + VD-FA/VB_12_ group (*p* = 0.008, *p* = 0.023) in the liver, while the mRNA expression of VDR was upregulated in the VDD + FA/VB_12_ group (*p* = 0.003) and VDD + VD-FA/VB_12_ group (*p* = 0.005). The mRNA expression of CYP27A1 and CYP27B1 were upregulated in the VDD group (*p* < 0.001), VDR had a decreasing trend compared with the CON group in the brain; while the mRNA expression of CYP27A1 (*p* = 0.002, *p* < 0.001) and CYP27B1 (*p* = 0.008, *p* < 0.001) were downregulated, VDR (*p* = 0.044, *p* = 0.010) was upregulated in the VDD + VD group and VDD + VD-FA/VB_12_ group compared with the VDD group in the brain. These results revealed that the mRNA expression of CYP27A1 and CYP27B1 were upregulated, while VDR was downregulated in the liver and brain of mice under vitamin D deficient conditions. Intervention using vitamin D alone or in combination with B vitamins may reverse this situation.

**Figure 7:**
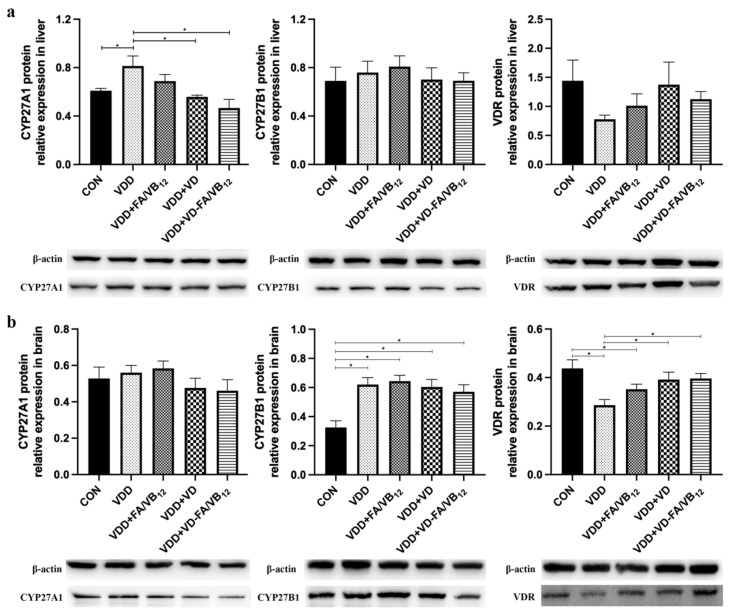
The protein expression of CYP27A1, CYP27B1, and VDR in liver and brain. (**a**) CYP27A1, CYP27A1and VDR protein in livers; (**b**) CYP27A1, CYP27A1and VDR protein in brains. CON: control; VDD: vitamin D-deficient diet; VDD + FA/VB_12_: vitamin D-deficient diet plus folic acid/vitamin B_12_ supplement; VDD + VD: vitamin D-deficient diet plus vitamin D supplement; VDD + VD-FA/VB_12_: vitamin D-deficient diet plus vitamin D and folic acid/vitamin B_12_. All data are presented as the mean ± SEM (*n* = 4). A two-sided *p* < 0.05 was considered statistically significant.

In order to clarify the effect of vitamin D deficiency and intervention on the above indicators, their expression on protein levels in livers and brains was also detected using western blot. Details of the results are shown in Figure 7. Compared with the CON group, the protein expression levels of CYP27A1 (*p* = 0.021) were upregulated, and CYP27B1 protein expression had a similar trend in the liver. Compared with the VDD group, the protein expression of CYP27A1 was downregulated in the VDD + VD group (*p* = 0.006) and VDD + VD-FA/VB_12_ group (*p* = 0.001) in the liver. The protein expression CYP27B1 was upregulated in the VDD group (*p* < 0.001) and VDR was downregulated compared with the CON group in the brain, CYP27A1 also had an increased expression trend in the VDD group. The protein expression of VDR was upregulated in the VDD + VD group (*p* = 0.013) and VDD + VD-FA/VB_12_ group (*p* = 0.010) compared with the VDD group in the brain. Overall, the protein expression of CYP27A1, CYP27B1, and VDR followed a similar trend to mRNA expression.

## 4. Discussion

Vitamin D deficiency is a widespread nutritional deficiency. Among the elderly Chinese population, 34.1% of men and 44.0% of women have vitamin D deficiency according to the analysis of the China national nutrition and health survey (CNNHS) [2]. Vitamin D deficiency has been thoroughly investigated to be a risk factor for AD, however, the roles of vitamin D deficiency in the pathology of AD and the underlying mechanism remain largely unexplored [28]. In this study, we found that vitamin D deficiency was associated with long-term spatial memory and short-term memory impairment in mice. A previous animal study also demonstrated that vitamin D-deficient diet-fed (<5 IU/kg vitamin D_3_) mice exhibited impaired spatial learning and memory ability in 6-month-old APP/PS1 mice, suggesting that vitamin deficiency could accelerate cognitive impairment [5]. The results were also in accordance with another research study showing that vitamin D-deficient diet-fed (<25 IU/kg vitamin D_3_) mice could induce impairment in learning and memory ability, and longer latency to find the platform [29]. In male BALB/c mice, it has been found that vitamin D deficiency was related to hippocampal-dependent spatial learning impairment [30]. These studies strongly suggest that vitamin D deficiency is a critical risk factor for cognition decline.

Therefore, intervention in vitamin D deficiency may play an important role in preventing AD at the early stages. In the present study, the results showed that vitamin D supplementation in vitamin D-deficient mice could reverse the impaired ability of learning and memory. Moreover, we found that vitamin D, folic acid, and vitamin B_12_ co-supplementation may have the same or even better effect than vitamin D supplementation alone. An animal study observed that vitamin D deficiency damaged neurogenesis both in wild-type and 5XFAD transgenic mice, and high vitamin D supplementation was efficient in improving learning and memory ability and neurogenesis in the brain [31]. Previously, the Dutch B-PROOF multi-center intervention study found that combined supplementation of vitamin D, folic acid, and vitamin B_12_ group slowed down the decline of overall cognitive function to a certain extent, in addition to preventing osteoporosis [32]. Although this cognitive improvement effect is relatively limited, it also suggested an interaction between vitamin D, folic acid, and vitamin B_12_, and it was also confirmed in this study. Thus, the co-supplementation of vitamin D, folic acid, and vitamin B_12_, may have a better positive effect than vitamin D supplementation alone in cognitive function, and it should be further explored and verified in population research.

Vitamin D can convert cholecalciferol to 25(OH)D by the enzyme CYP27A1, while, 25(OH)D is further hydroxylated by CYP27B1 to produce the active vitamin D metabolite [33]. Vitamin D receptor (VDR), a nuclear receptor, is the main factor for vitamin D playing biological effects. In the present study, we found that vitamin D deficiency resulted in higher expression of CYP27A1 and CYP27B1, both of which were downregulated when Vitamin D, folic acid, and vitamin B_12_ were supplemented in the diets. In contrast, expression of VDR was downregulated in mice consuming a vitamin D-deficient diet and upregulated after vitamin D, folic acid, and vitamin B_12_ supplementation. A previous study using high-fat diet animal models found that vitamin D deficiency could also upregulate the expression of CYP27A1, and vitamin D supplements reversed it, which suggests that there may be an interaction between high cholesterol and vitamin D [34]. CYP27B1 belongs to the same family of CYP27A1, it has been found that vitamin D-deficient mice could significantly upregulate the expression of CYP27B1 in C57BL/6J mice as well, which was positively correlated to CYP27A1 expression [35]. An animal study demonstrated that vitamin D-deficient diet-fed (<200 IU/kg vitamin D_3_) mice performed downregulated expression of VDR, then further reduced the biological effects of vitamin D, consistent with our results [36]. Therefore, vitamin D metabolic disorder would induce abnormal expression of its metabolic enzymes and may further affect its physiological process.

Besides, as a cholesterol-oxidized metabolic enzyme, CYP27A1 is also critical for the synthesis of 27-OHC, which is one of the most important risk factors in the pathology of AD. A meta-analysis indicated that the increased levels of 27-OHC may be a potent biomarker for the evaluation or recognition of MCI and AD [7]. Given vitamin D and 27-OHC have been confirmed to be associated with AD, we explored whether vitamin D deficiency affects AD by regulating the expression of CYP27A1. This study showed that vitamin D deficiency resulted in increased levels of 27-OHC, which was reversed by vitamin D supplements alone or in combination with B vitamins. An animal study found that vitamin D supplementation significantly downregulated the expression of CYP27A1 in adipocytes, indicating that vitamin D supplementation may reduce the expression of CYP27A1 [34]. Meanwhile, a pilot breast cancer trial in the USA observed a significantly increased level of 25(OH)D and a decreased level of 27-OHC in high-dose vitamin D intervened subjects compared with that in low-dose supplied subjects, and 25(OH)D was inversely correlated to 27-OHC level [37]. Results of both studies suggested that vitamin D supplementation may decrease circulating 27-OHC by suppressing CYP27A1.

25(OH)D is often measured in serum to determine vitamin D status. In the presence of vitamin D deficiency, the level of 25(OH)D could decrease significantly, which was further confirmed in the present study. A large-scale aging cohort study found that the aging population has a high burden of vitamin D, folic acid, and vitamin B_12_ deficiencies, which induced potential negative consequences on cognitive function [38]. Therefore, we supplemented vitamin D, folic acid, and vitamin B_12_ in vitamin D-deficient mice and showed that co-supplementation produced better results in improving cognition and increasing serum levels of SAM. SAM was found to decrease in the population with AD in a cross-sectional study [39]. An animal study performed in B vitamin-deficient diet-fed TgCRND8 and wild-type mice found that SAM supplementation reduced hyperhomocysteinemia and spatial memory impairment induced by B vitamin deficiency [40]. A randomized controlled trial assessed the effect of a folic acid supplement on cognitive function in AD patients, indicating that folic acid could be beneficial for patients with AD by regulating inflammation reactions [41]. Another in-depth study showed that vitamin B_12_ might alleviate bioenergetic defects and oxidative stress, and delay beta-induced paralysis by regulating the methionine/SAM cycle [42]. Thus, as modifiable targets, both SAM and 27-OHC may be responsible for cognitive impairment due to vitamin D deficiency to some extent. More research focusing on the effects and underlying mechanisms of vitamin co-supplementation on AD is still necessary.

**Figure 8:**
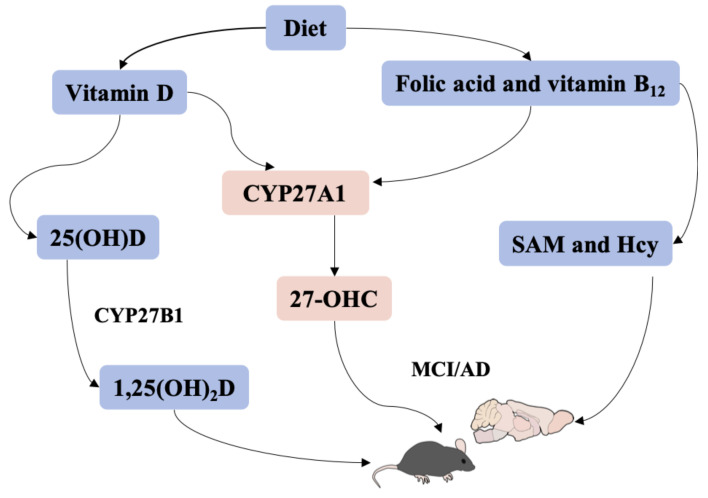
A graphic abstract.

To the best of our knowledge, despite single vitamin D, folic acid, and vitamin B_12_ having been widely considered as potential treatment strategies for cognitive impairment, no study has been conducted to investigate their potential interactions, especially in an animal model [43,44,45]. Herein, we observed the effect of vitamin D, folic acid, and vitamin B_12_ co-supplementation on learning and memory ability in vitamin D-deficient mice. The results demonstrated that supplementation with vitamin D, folic acid, and vitamin B_12_ alone or together was efficient in improving learning and memory ability in vitamin D-deficient mice, and co-supplementation may be more effective. Besides, the current study provided a new perspective that CYP27A1 may be a mediator for vitamin D, 27-OHC, SAM, and learning and memory ability. However, whether CYP27A1 plays a decisive role in vitamin D-related pathology of AD needs to be verified in further studies. A graphic abstract is shown in Figure 8.

## 5. Conclusions

In summary, the present study indicated that vitamin D deficiency was closely associated with learning and memory impairment in mice, probably in part by inducing 27-OHC metabolism disorders through upregulating the expression of CYP27A1. A combined supplementation of vitamin D, folic acid, and vitamin B_12_ may collectively exert effects on learning and memory ability by lowering the expression of CYP27A1 and regulating the levels of 27-OHC and SAM. These findings will provide further insights into the involvement of vitamin D, folic acid, and vitamin B_12_ co-supplementation in cognitive function, and the potentially significant role of the key factor CYP27A1 in the pathogenesis of MCI and AD. In addition, this study could also be referenced in the development of strategies to address vitamin D deficiency and prevent or mitigate adverse consequences. 

## Data Availability

The datasets during and/or analyzed during the current study are available from the corresponding author upon reasonable request.

## Supplementary Materials

The following supporting information can be downloaded at: https://www.mdpi.com/article/10.3390/nu15010132/s1, Table S1: Formulation of Control diet and Vitamin D-deficient diet; Table S2: The primer sequences used for qRT-PCR.

## Abbreviations

27-hydroxycholesterol (27-OHC); 25-hydroxyvitamin D 25(OH)D; Alzheimer’s Disease (AD); homocysteine (Hcy); Frequency discrimination index (FDI); Mild cognitive impairment (MCI); S-Adenosylmethionine (SAM); Time discrimination index (TDI); Vitamin D-deficient diet (VDD).
