# Supplementary material for: Vitamin D, Folic Acid and Vitamin B12 Can Reverse Vitamin D Deficiency-Induced Learning and Memory Impairment by Altering 27-Hydroxycholesterol and S-Adenosylmethionine
Source: Nutrients. 2022 Dec 27;15(1):132. doi: 10.3390/nu15010132 (PMC9824694; doi:10.3390/nu15010132)
Supplement: Supplementary file 1 [file nutrients-15-00132-s001.zip › nutrients-2059542-supplementary.pdf]

nutrients

Article

Vitamin D, Folic Acid and Vitamin B12 Can Reverse Vitamin D Deficiency-Induced Learning and Memory Impairment by Altering 27-Hydroxycholesterol and S-Adenosylmethionine

Supplementary Materials

Supplementary Table S1. Formulation of Control diet and Vitamin D-deficient diet.

Ingredients	Control Diet		Vitamin D-Deficient Diet	
	gm	kcal	gm	kcal
Casein, “Vitamin-Free” Test	0	0	140	560
Casein, regular	140	560	0	0
L-Cysteine	1.8	7.2	1.8	7.2
Dextrose	0	0	595,.692	2383
Corn Starch	495,692	1983	0	0
Sucrose	100	400	100	400
Cellulose	50	0	50	0
Maltodextrin	125	500	125	500
Soybean Oil	40	360	40	360
Mineral Mix	35	0	35	0
Vitamin mix	10	40	0	0
Vitamin mix (*w/o* Va,d)	0	0	10	40
Vitamin A (500,000 IU/gm)	0	0	0.008	0
Choline Bitartrate	2.5	0	2.5	0
t-Butylhydroquinone	0.008	0	0.008	0
Total	1000	3850	1000.008	3850

Supplementary Table S2. The primer sequences used for qRT-PCR.

Gene	Forward Sequence (5′–3′)	Reverse Sequence (5′–3′)
CYP27A1	AACAAGGACTTTGCCCACATG	GCGCAGGGTCTCCTTAATCA
CYP27B1	AGATACGCTAGTCTCCCTATGT	GCACTTCAAAATGGGTCAAGAT
VDR	TCAAACTCTGATCTGTACACCC	TGGATGCTGTAACTGACAAGAT
